# Clindamycin-induced acute generalized exanthematous pustulosis: A case report

**DOI:** 10.1097/MD.0000000000020389

**Published:** 2020-05-22

**Authors:** Kumpol Aiempanakit, Benjawan Apinantriyo

**Affiliations:** a Division of Dermatology, Department of Internal Medicine, Faculty of Medicine, Prince of Songkla University; b Hematology Unit, Medical Specialty Center, Bangkok Hospital Hat Yai, Bangkok Dusit Medical Services, Hat Yai, Songkhla, Thailand.

**Keywords:** acute generalized exanthematous pustulosis, clindamycin, pus-fluid levels, pustular drug eruptions, Sneddon–Wilkinson disease

## Abstract

**Rationale:**

Acute generalized exanthematous pustulosis (AGEP) is a severe pustular cutaneous adverse drug reaction. Sterile, non-follicular pustules overlying the erythematous skin characterize this reaction.

**Patient concerns:**

A 30-year-old Asian women presented with sterile, non-follicular lesions with pus-fluid levels on her back 2 days after taking clindamycin. Skin biopsy revealed a spongiotic change in the epidermis with a focal subcorneal pustule and perivascular eosinophil and lymphocyte infiltration.

**Diagnosis:**

Clindamycin-induced AGEP.

**Interventions:**

We discontinued clindamycin treatment and prescribed systemic corticosteroids.

**Outcomes:**

The pustule stopped spreading within 1 day and the rash improved within 2 days.

**Lessons:**

AGEP is a pustular cutaneous adverse drug reaction that can appear with pus-fluid levels, clinically mimicking Sneddon–Wilkinson disease. The differentiation between both conditions is a history of drug use, characteristic skin lesions and histopathology.

## 1 Introduction

Acute generalized exanthematous pustulosis (AGEP) is a severe pustular cutaneous adverse reaction, commonly caused by medications. It is characterized by acute pinpoint, sterile, non-follicular pustules overlying the erythematous skin.^[1–3]^ Other cutaneous presentations may occur, such as facial edema, purpura, atypical target-like lesions, and blisters. The systemic manifestations are acute onset of fever (>38°C), mild and transient involvement of the internal organs, such as the liver and kidneys.^[1]^ Here, we report a case of an Asian women with clindamycin-induced AGEP with cutaneous lesions mimicking Sneddon–Wilkinson disease (SWD). To the best of our knowledge, a clinical case of AGEP with pus-fluid levels has not yet been reported.

## 2 Case report

**Figure 1:**
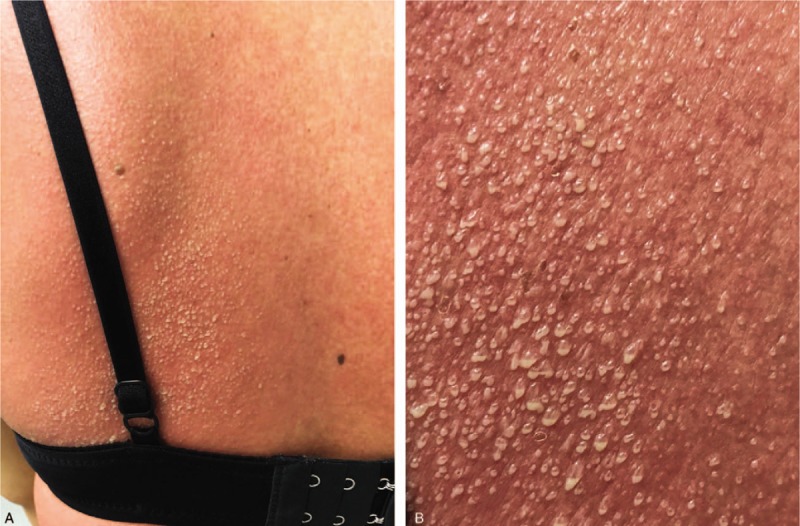
Clinical photographs. (A) Pus-fluid levels on erythematous patch predominate on the back. (B) Close-up photograph showing pus accumulating in the lower half with overlying clear fluid.

A 30-year-old Asian woman presented with a 2-day history of generalized itchy erythematous rash. She reported that she had fever and sore throat 4 days before. She sought medical attention at a private hospital and was diagnosed with acute pharyngitis. She was administered clindamycin, paracetamol, codeine phosphate, and guaifenesin. Two days after treatment initiation with clindamycin (a total of 5 doses), she developed an erythematous pruritic rash on the extremities and trunk. She denied any personal history of drug or food allergy. Physical examination revealed an erythematous patch on her back with non-follicular pustules and pus-fluid levels, wherein the pus accumulates in the lower half of the pustule with an overlying clear fluid (Fig. 1), and generalized erythematous macules, papules, and patches on her arms, chest, and abdomen. On admission, she had low-grade fever and other vital signs were normal. There was no ocular or mucosal involvement and lymphadenopathy.

**Figure 2:**
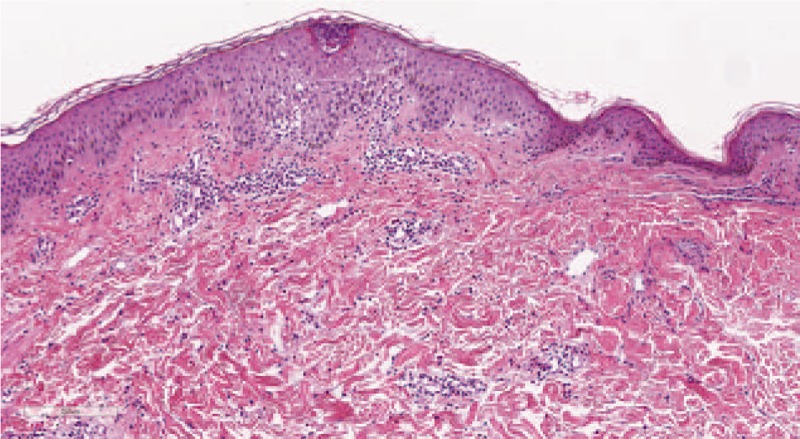
Dermatopathology studies showing spongiosis of the epidermis with a focal subcorneal pustule and superficial perivascular eosinophil and lymphocyte infiltration (hematoxylin–eosin stain, original magnification ×10).

Laboratory tests revealed the following: hemoglobin level 13.2 (12.0–16.0) g/dl, hematocrit level 39.3 (36.0–48.0)%, white cell count 12.9 × 10^9^ (4.0–10.0)/L (neutrophils 87%, lymphocytes 10%, eosinophils 1%, and monocytes 2%), platelet count 299 × 10^9^ (1500–450)/L, and serum creatinine level 0.59 (0.51–0.95) mg/dl. The liver function test results were normal and the anti-nuclear antibody test results were negative. For skin biopsy, a skin sample from the back was taken. Histopathology revealed the spongiotic change in the epidermis with a focal subcorneal pustule, superficial and deep perivascular and periadnexal eosinophil and lymphocyte infiltration, and no definite vasculitis (Fig. 2). Periodic acid–Schiff–diastase and Gomori methenamine silver staining results were negative for fungi. The pus gram staining result was negative for bacteria. The blood and pus culture results were also negative.

The patient was diagnosed with AGEP due to the characteristic rash and the skin biopsy results. Clindamycin was the probable cause, assessed using the Jones algorithm.^[4]^ Treatment with clindamycin was discontinued and treatment with systemic, topical steroids (intravenous dexamethasone, 16 mg/d for 3 days and topical 0.25% desoximetasone cream) and antihistamines (hydroxyzine 20 mg/d, chlorpheniramine 20 mg/d, and fexofenadine 180 mg/d) was initiated. The pus-fluid levels had stopped spreading within 1 day and the maculopapular rash alleviated within 2 days. She was discharged after 3 days and was prescribed oral prednisolone 30 mg/d for 4 days. At the 1-week follow-up, she was free of skin eruptions.

## 3 Discussion

AGEP is a rare but severe adverse reaction to some medications. AGEP has been associated with various etiologies, such as use of antibiotics, omeprazole, non-steroidal anti-inflammatory drugs, and herbs; viral infection; and mercury and ultraviolet light exposure.^[1–3]^ The main cause of AGEP is antimicrobial exposure, particularly beta-lactams, macrolides, and lincosamide antibiotics.^[2]^ Other antimicrobials, such as quinolones, tetracyclines, and sulfonamides, and oral antifungal agents, such as terbinafine and itraconazole, have also been known to cause AGEP.^[1–3]^

In this case, the patient was administered clindamycin for acute pharyngitis. Clindamycin is an antibiotic of the lincosamide group with a primarily bacteriostatic action against gram-positive aerobes and a broad spectrum of anaerobic bacteria. Clindamycin binds to the 50S subunit of the bacterial ribosome and inhibits the early stages of protein synthesis.^[5]^ Previous studies have reported that the cases of clindamycin-induced AGEP have been increasing.^[5–13]^

The pathophysiology of AGEP is a delayed-type hypersensitivity to a specific drug. After drug exposure, cluster of differentiation (CD) 4 and CD8 lymphocytes are activated, resulting in the migration of T cells to the skin. The drug-specific CD8 T-cells consume perforin, granzyme B, and Fas ligand, affecting the keratinocytes apoptosis and resulting in epidermal vesicle formation. The systemic involvement in AGEP, such as hepatitis or renal insufficiency, may be due to the circulating interleukin (IL)-17 and IL-22.^[1,5]^ Nevertheless, the exact mechanism is uncertain.

The clinical characteristics of AGEP are diffuse, pinpoint, sterile, non-follicular pustules overlying the erythematous skin. They appear 24 to 48 hours after the initiation of the implicated drug.^[2]^ The rash predominates on the trunk and intertriginous regions. Systemic signs include leukocytosis with neutrophilia, fever, elevated liver enzyme levels, and renal insufficiency.^[1]^ The differential diagnoses of the pustular rash include pustular psoriasis, subcorneal pustular dermatosis (SWD), and pustular vasculitis.

SWD is a rare, chronic, recurrent pustular dermatosis. The clinical characteristics involve multiple small pustules with pus-fluid levels on the dependent area, whereby pus accumulates in the lower half with an overlying clear fluid.^[14]^ In this case report, the patients had skin lesions like SWD, showing pus-fluid levels. To the best of our knowledge, there are no previous studies reporting this clinical presentation in AGEP. Based on the pathophysiology of AGEP, the vesicle may appear in the very early phase. If the patient adopts a dependent position, the pus, which mainly consists of neutrophils, accumulates in the lower half of the pustule due to gravity. The differentiation between these 2 conditions is including a history of medication usage before developing the lesions, a clinical course, some clinical characteristics, and histology.^[14,15]^ AGEP almost always has a history of drug use. Its histology shows spongiosis with necrotic keratinocytes and eosinophil infiltration. While, SWD has a chronic and recurrent course, which may present annular, circinate, or serpiginous patterns.

The typical histopathology of AGEP shows spongiform subcorneal pustules, often-marked edema of the papillary dermis, and perivascular infiltration with neutrophils and exocytosis of some eosinophils.^[2]^ Discontinuing the causative medication is necessary for treatment.

In conclusion, AGEP is a severe cutaneous adverse reaction to medications, especially antibiotics. Clindamycin is an antibiotic that has been reported to cause AGEP. The patient was diagnosed with clindamycin-induced AGEP, and the clinical characteristics mimicked SWD. Furthermore, pustular lesions in AGEP can be present with pus-fluid levels. Further clinical studies on AGEP, where patients are carefully examined for these signs, might increase our understanding of the illness.

## Author contributions

**Conceptualization:** Kumpol Aiempanakit, Benjawan Apinantriyo.

**Data curation:** Kumpol Aiempanakit, Benjawan Apinantriyo.

**Investigation:** Kumpol Aiempanakit, Benjawan Apinantriyo.

**Methodology:** Kumpol Aiempanakit.

**Supervision:** Kumpol Aiempanakit.

**Validation:** Kumpol Aiempanakit, Benjawan Apinantriyo.

**Writing – original draft:** Kumpol Aiempanakit, Benjawan Apinantriyo.

**Writing – review & editing:** Kumpol Aiempanakit, Benjawan Apinantriyo.
